# Correction to: The apple REFPOP—a reference population for genomics-assisted breeding in apple

**DOI:** 10.1093/hr/uhac272

**Published:** 2022-11-01

This is a correction to: Michaela Jung, Morgane Roth, Maria Jos Aranzana, Annemarie Auwerkerken, Marco Bink, Caroline Denanc, Christian Dujak, Charles-Eric Durel, Carolina Font i Forcada, Celia M Cantin, Walter Guerra, Nicholas P Howard, Beat Keller, Mariusz Lewandowski, Matthew Ordidge, Marijn Rymenants, Nadia Sanin, Bruno Studer, Edward Zurawicz, Franois Laurens, Andrea Patocchi, Hlne Muranty, The apple REFPOP—a reference population for genomics-assisted breeding in apple, Horticulture Research, Volume 7, 2020, 189, https://doi.org/10.1038/s41438-020-00408-8

An error resulting from an incorrect match of datasets produced using the Illumina Infinium® 20K SNP genotyping array and the Affymetrix Axiom® Apple 480K SNP genotyping array has been discovered in the imputed genomic dataset of the apple REFPOP. The discovered error was corrected, and statistical analyses were repeated using the corrected imputed genomic dataset. Change in average predictive ability was found for floral emergence from the original 0.57 to the corrected 0.58 and for harvest date from 0.75 to 0.77 using the RR-BLUP model. Correctness of the published results of GWAS was supported when marker-trait associations in the same genomic regions as reported here were rediscovered by Jung et al. (2022) *Horticulture Research* (Supplementary Table 3) using GWAS that was based on the corrected imputed genomic dataset.

These details have been corrected only in this correction notice to preserve the published version of record.

